# Differential Serum Peptidomics Reveal Multi-Marker Models That Predict Breast Cancer Progression

**DOI:** 10.3390/cancers16132365

**Published:** 2024-06-27

**Authors:** Adhari AlZaabi, Stephen Piccolo, Steven Graves, Marc Hansen

**Affiliations:** 1 Department of Human and Clinical Anatomy, Sultan Qaboos University, 35, Muscat 123, Oman; 2 Department of Physiology and Developmental Biology, Brigham Young University, Provo, UT 84602, USA; 3 Department of Biology, Brigham Young University, Provo, UT 84602, USA; 4 Department of Biomedical Informatics, University of Utah, Salt Lake City, UT 84112, USA; 5 Department of Chemistry and Biochemistry (Emeritus), Brigham Young University, Provo, UT 84606, USA; swgraves@chem.byu.edu; 6 Magellan Bioanalytics, Inc., Pleasant Grove, UT 84062, USA; biotechmarc@gmail.com

**Keywords:** breast cancer, metastasis, cancer progression, biomarker, serum, peptidomics, proteomics, machine learning

## Abstract

**Simple Summary:**

This manuscript details an investigation into the potential of mass spectrometry-based serum biomarker discovery for differentiating between early and late-stage breast cancer patients. The study employs a ‘feature-first’ approach, focusing on MS1 scan spectra differences and applies both traditional and computational methods to analyze these differences. The traditional method involves manual data alignment and validation, while the computational approach utilizes machine learning to assess biomarker relevance and validate predictive models. A key finding includes the identification of a peptide fragment of fibrinogen α chain as a biomarker exclusive to early-stage breast cancer.

**Abstract:**

Here, we assess how the differential expression of low molecular weight serum peptides might predict breast cancer progression with high confidence. We apply an LC/MS-MS-based, unbiased ‘omics’ analysis of serum samples from breast cancer patients to identify molecules that are differentially expressed in stage I and III breast cancer. Results were generated using standard and machine learning-based analytical workflows. With standard workflow, a discovery study yielded 65 circulating biomarker candidates with statistically significant differential expression. A second study confirmed the differential expression of a subset of these markers. Models based on combinations of multiple biomarkers were generated using an exploratory algorithm designed to generate greater diagnostic power and accuracy than any individual markers. Individual biomarkers and the more complex multi-marker models were then tested in a blinded validation study. The multi-marker models retained their predictive power in the validation study, the best of which attained an AUC of 0.84, with a sensitivity of 43% and a specificity of 88%. One of the markers with *m*/*z* 761.38, which was downregulated, was identified as a fibrinogen alpha chain. Machine learning-based analysis yielded a classifier that correctly categorizes every subject in the study and demonstrates parameter constraints required for high confidence in classifier output. These results suggest that serum peptide biomarker models could be optimized to assess breast cancer stage in a clinical setting.

## 1. Introduction

Metastasis, the colonization of distant tissues with cancer cells, is associated with a poor prognosis for all tumors [1]. Adjuvant therapy is used to eradicate escaped tumor cells and reduce the risk of recurrence. Accurately determining metastatic and pre-metastatic load with high sensitivity is required to personalize adjuvant treatment [2]. Adjuvant therapy remains overused because there is no non-invasive and accurate method to detect, measure, or monitor the colonization of non-local sites, such as lymph nodes, with cancer cells in earlier disease stages. Invasive, painful biopsies of potentially involved sites only detect tumor cell infiltration in the biopsy sample. Meanwhile, radiological studies are technically difficult and suffer from low sensitivity and specificity for smaller masses [3].

Researchers propose that communication between primary and secondary tumors occurs through circulating proteins, nucleic acids, and lipids [2,4]. It follows that the analysis of serum samples of patients with metastatic and non-metastatic breast cancer will reveal biomarkers that indicate the presence of metastatic lesions. Since serum is readily accessible for biomarker investigation, it is clinically appealing [5].

Serum-based, disease-specific diagnostic molecules are generally small peptides (under 50,000 Da) of low abundance [6,7]. Such peptides are produced either by cellular synthesis or by the controlled breakdown of larger intracellular proteins [8,9]. Previously considered cellular waste, such molecules have been found to reflect global physiological and pathophysiological states [10]. A technical challenge in accessing these molecules is that high abundance serum proteins of low information value mask and sequester these smaller, low-abundance, high information species. 

Here, we seek to assess the potential of low-abundance serum species as biomarkers for breast cancer lymph node infiltration. Where we assess the potential of finding novel serum biomarkers that, in combination, can accurately predict breast cancer stage from only a small set of clinical samples. We employ both a standard approach backed by classical statistics and a machine learning-based approach that uses algorithmic tests to determine confidence.

## 2. Materials and Methods

### 2.1. Serum Samples

In the discovery study, serum samples (Proteogenex, Ingelwood, CA, USA) taken from patients with stage I or III breast cancer were selected for matches based on race, age, and tumor subtype. For the confirmatory and validation studies, serum samples from patients with stage I or III breast cancer (Conversent Bio, Alameda, CA, USA) included a wider variety of sample types with less matching based on demographic factors such as treatment regimens, race, or age (Supplementary Table S1). Samples used in the confirmation and validation studies were blinded by an arbiter and randomly divided into training (n = 24) and testing (n = 15) sets, each with an approximately equal number of stage I and III samples.

### 2.2. Sample Processing

To separate low-abundance, high-information species from high-abundance serum proteins, we employed a selective precipitation approach used in prior studies [10,11,12,13]. Acetonitrile was added to samples to a final ratio of 2:1 (*v*:*v*, acetonitrile:serum). Samples were subjected to vortex mixing and then left at 25 °C for 30 min. Precipitates were removed by centrifugation at 13,400 rpm for 10 min at 25 °C and protein content determined using the BCA Protein Assay (Thermo Scientific, Waltham, MA, USA). An aliquot containing 4 μg apparent protein was concentrated to a final volume of 10 μL using a CentriVap Concentrator (Labconco Corporation, Kansas City, MO, USA) and then diluted with an equal volume (10 μL) of 88% formic acid.

### 2.3. Chromatographic Separation

Reversed-phase capillary liquid chromatography (cLC) involved an LC Packings Ultimate Capillary (HPLC) pump system with a Dionex FAMOS autosampler (Sunnyvale, CA, USA). The cLC columns included a 1 mm (16.2 μL) dry-packed MicroBore guard column (IDEX Health and Science, Oak Harbor, WA, USA) coupled with a 15 cm × 250 μm i.d capillary column, slurry-packed in-house with Poros R1 reversed-phase medium (Applied Biosystems, Foster City, CA, USA). The elution gradient mixed an aqueous phase (2% acetonitrile and 0.1% formic acid) and an organic phase (98% acetonitrile and 0.1% formic acid).

### 2.4. Mass Spectrometry

The cLC system was interfaced to an Agilent 6530 Accurate-Mass Q-TOF LC/MS system (Santa Clara, CA, USA) in positive ion mode. The scans were collected at 8 spectra/s, and the mass spectra were collected within a range of *m*/*z* 500 to *m*/*z* 2500. When conducting fragmentation studies, scans were collected at 1 spectra/s. Agilent MassHunter Qualitative Analysis B.06.00 software was used to extract ion intensities. To counter batch effects [14,15], specimens from both comparison groups were part of each batch and further applied the ComBat algorithm [16] in the confirmatory study. Elution profiles were normalized by aligning peaks of established time markers [11,12,13] and initially compared by visual inspection. Ion counts of differentially expressed peaks (>50% greater or less by eye) were extracted and normalized by calculating variation from the mean across all samples for the same marker. Normalized intensities were statistically evaluated using Student’s *t*-test.

### 2.5. Statistical Analysis

We used machine learning algorithms to derive multivariate predictive models. We applied two-fold cross-validation to the training data and used a forward-selection approach to identify combinations of markers that were most predictive of cancer stage. First, we identified individual markers that attained an area under the receiver operating characteristic curve, or AUC, of greater than 0.65. We then evaluated all pairwise combinations of these to identify those sets that attained an AUC > 0.70. We sequentially added additional markers to each multi-marker model to reveal which markers increased the AUC value at each step. We used AUC thresholds of 0.75, 0.85, 0.90, and 0.95 for 3-way, 4-way, 5-way, and 6-way marker combinations, respectively. We applied multiple classification algorithms to the training data, including support vector machines (SVM), random forests, k-nearest neighbors, Naive Bayes classifier, and logistic regression. We wrote custom scripts using the R statistical software and the Python (version 3.7) programming language (https://python.org, accessed 26 April 2017). For the classification analysis, we used the mlr package [17].

### 2.6. Peak Identification

Markers were subjected to tandem mass spectrometry with collision-induced dissociation (CID). Tandem MS studies with nitrogen and/or argon were conducted at several energies to increase fragment ion coverage, and these individual MS2 spectra were summed. The composite fragmentation spectrum was inspected visually for incorrectly assigned charge states, and these were corrected for submission to a Mascot database search to determine amino acid sequences.

### 2.7. Machine Learning

In total, 39 mass spec output files, comprising mass spectra derived from 20 early-stage (stage I) and 19 late-stage (stage III) human serum samples, were converted to MZml format for analysis using COMPASS software, version 1.1 (Magellan Bioanalytics, Pleasant Grove, UT, USA). Patient data surrounding the samples was supplied in an assembly file. To compare results achieved with correctly assigned data to those achieved by overfitting random noise in the large datasets, a mock assembly file was generated and subjected to the same analysis as for the correctly assigned dataset. Subject breast cancer stage was switched for 10 early-stage and 9 late-stage subjects in the mock assembly file. Relevant COMPASS analytical parameters used in the analysis are outlined in the results section.

## 3. Results

### 3.1. Biomarker Discovery

For the standard workflow, we conducted separate biomarker discovery, confirmation, and validation studies. In this workflow, we sought to identify differentially expressed biomarkers by manual alignment and comparison of the mass spectra. We selected biomarkers whose differential expression yielded a *p*-value below 0.05 when generated using a Student’s *t*-test, accepting that the small study and large number of features would generate many ‘statistically significant’ biomarkers that would not survive subsequent confirmation. The confirmed biomarkers and their expression in the confirmation set were used to train a machine learning model and tested against an additional set of validation samples.

Our discovery study was designed to identify serum biomarkers that are differentially expressed in stage I and III breast cancer patients. To minimize confounding factors, serum samples were selected from patients with few comorbidities who had similar tumor subtypes and had received similar treatment regimens as much as possible. Similarly, samples were chosen from patients of similar age and race. Six stage I and six stage III breast cancer patient serum samples were subjected to acetonitrile precipitation to remove large and abundant proteins, leaving samples with highly diverse low-molecular-weight species that were analyzed by mass spectrometry. 

Samples yielded complex mass spectra with over 10,000 distinct peaks, which were aligned by reference to a set of established time markers for comparative analysis. Visual scanning of the spectra was performed to identify peaks with possible differential expressions between the sample sets. The visual inspection utilized here is designed to ensure a comprehensive evaluation of potential peaks, which was reported earlier [11,13]. This includes standardized mass spec data processing criteria to reduce bias and improve data integrity. For standardization, species co-eluting with comparable intensities across cases and controls were selected as reference peaks. A peak qualified as a reference peak if it met the following criteria: it must elute within the same time interval as the peak of interest, be present in every specimen, and have a mass-to-charge ratio very close to that of the candidate peak. Additionally, the reference peak needed to demonstrate consistent quantitative comparability between cases and controls, as assessed initially through visual inspection followed by extraction via machine software. The intensities of these reference peaks were then determined and utilized for the normalization of potential biomarkers.

Numeric ion counts for each candidate peak were extracted, and statistical evaluation of the differences between the total mean normalized intensities was performed (Table 1). This provided 65 candidate biomarkers with statistically significant (*p* < 0.05) differential expression. Another 21 biomarkers were differentially expressed at near-statistical significance (0.05 < *p* < 0.10). Among the significant markers (n = 65), 9 were up-regulated in serum from patients with metastatic disease, while 56 were down-regulated. Thus, this discovery small group study demonstrated that ’omics’ analysis of highly diverse, low-abundance serum species yielded differentially expressed biomarkers that might correctly classify disease state.

### 3.2. Biomarker Confirmation Study

To confirm the differential expression of the putative biomarker species identified in our discovery study, we assessed their expression in a second confirmatory study composed of a larger sample set. Here, we were less stringent about controlling for tumor subtype, patient age and race, and comorbidity. Twenty serum samples from stage I and nineteen serum samples from stage III breast cancer patients were processed and analyzed using cLC-MS instrumentation with the same methods used in the discovery study. Prior to sample processing, patient information was masked to prevent differences in handling, processing, instrumentation, or analysis. The resulting spectra were time aligned using endogenous time markers to align all samples for comparison, both within this sample set and with the spectra generated in the discovery study. The peak intensities of candidate biomarkers were quantified for 24 serum samples that were selected for us by the independent arbiter who masked the study and ensured an almost equal number of stage I and III samples in this set. This left another 15 samples, also masked and containing nearly equal numbers of stage I and III samples, for further evaluation in a subsequent biomarker validation study.

To determine the power of each candidate biomarker to predict disease state, we applied the random forests algorithm (using a two-fold cross-validation design) to the results after unmasking the confirmation set. The random forest algorithm was selected, as it yielded the best AUC scores when applied to the training set when compared to other machine learning models tested (Supplementary Table S2). Although two-fold cross-validation is less conventional than using a larger number of folds, we chose this option because our forward selection process was highly computationally intensive. This eliminated many candidate biomarkers, as expected. While 16 of the markers retained statistical significance in this data set, only 12 were confirmed to provide relatively high predictive accuracy with an AUC over 0.65 (Table 2). The best serum biomarker was *m*/*z* 497.26, with an AUC of 0.79, a sensitivity of 0.79, and a specificity of 0.64.

### 3.3. Multi-Marker Model Construction and Validation

The power of one specific biomarker to distinguish between similar patient types is limited. Combining data from multiple markers is more likely to predict tumor stage despite differences in tumor heterogeneity, patient tumor–immune interactions, and other variabilities [18]. We, therefore, constructed multi-marker models that combine the predictive power of single confirmed biomarkers from this study.

We constructed a series of multi-marker models and assessed their staging power. We used a forward-selection algorithm to identify biomarker combinations whose expression provided the best accuracy for the discovery and confirmatory study samples. The algorithm combined data from two or more biomarkers and assessed whether the predictive power of the combined data was increased compared to the data from either individual biomarker. Combinations with reduced predictive power were abandoned, while those with increased predictive power were retained and iteratively supplemented with data from additional single biomarkers and assessed again. Thus, the algorithm assessed all possible combinations, yielding five promising biomarker profiles (Table 3).

We then sought to validate the predictive power of these multi-marker models using the remaining 15 serum samples reserved for validation purposes in our final study. This masked set contained eight stage I serum and seven stage III breast cancer serum samples. We evaluated the power of the five multi-marker models using the random forests algorithm, which performed best in the discovery phase. This algorithm has been used broadly in biomedical studies because it typically results in relatively high accuracy and is more easily interpretable than many other classification algorithms [19,20]. As expected, accuracy levels in the validation set were lower than were obtained in the confirmation set. Model C, consisting of markers with *m*/*z* 458.25 (z = +2), *m*/*z* 497.26 (z = +1), *m*/*z* 585.3 (z = +1), *m*/*z* 722 (z = +2), and *m*/*z* 923.45 (z= +1), attained an AUC of 0.804, with a sensitivity of 0.429 and a specificity of 0.875 (Table 3). With a threshold score of 0.7, indicating stage III disease, this model has a high sensitivity in identifying true positives and average specificity. In contrast, a threshold score of 0.9 has a low sensitivity in identifying patients with stage III disease but high specificity. Thus, while the small validation study reveals a multi-marker model with a limited ability to determine disease stage without false positives, it holds promise in identifying a subset of stage I patients.

### 3.4. Biomarker Identification

We sought to determine the molecular identity of biomarkers that comprised the multi-marker models. However, peptide fragmentation and analysis are technically challenging for low-abundance species. As peptide bond strengths differ, some expected fragments are yielded only at very low abundance and are indistinguishable from noise. This results in incomplete b- or y-series that make identification difficult or impossible. Signal suppression is also problematic. Finally, some peaks could not be confidently identified because of peak overlap.

**Figure 1:**
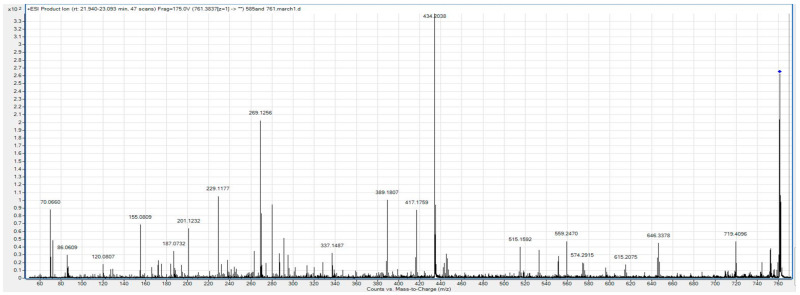
Fragmentation profile of the *m*/*z* 761.38 biomarker.

Despite these challenges, we were able to generate a partial fragmentation profile for the biomarker with *m*/*z* 761.38, whose peak is included in two of the best multi-marker models. Mass differences after fragmentation correspond most closely with an amino acid sequence of DLVPGNF (Figure 1).

BLAST reveals that this sequence is found within fibrinogen alpha chain (FGA) isomer 2, as well as within an un-named protein product (BAF83248.1). To confirm this sequence, we used the Fragment Ion Formula Calculator to provide a list of the expected b and y ion series that are expected in the ms/ms spectra of FGA (Table 4). These predicted ion series are all present in the fragmentation profile of *m*/*z* 761.38.

### 3.5. Machine Learning-Based Analysis

Given the limited predictive power of multimarker models based on labor-intensive data analysis and the risk of relying on a few select examples from a large dataset, we opted to try an algorithmic approach to biomarker identification. Here, the entire mass spectrum from each sample is subject to comparative analysis, allowing the identification of differentially expressed signals that are validated using machine learning. The same files used for the standard workflow were used in this workflow, so nothing about sample processing or instrumentation changed.

**Figure 2:**
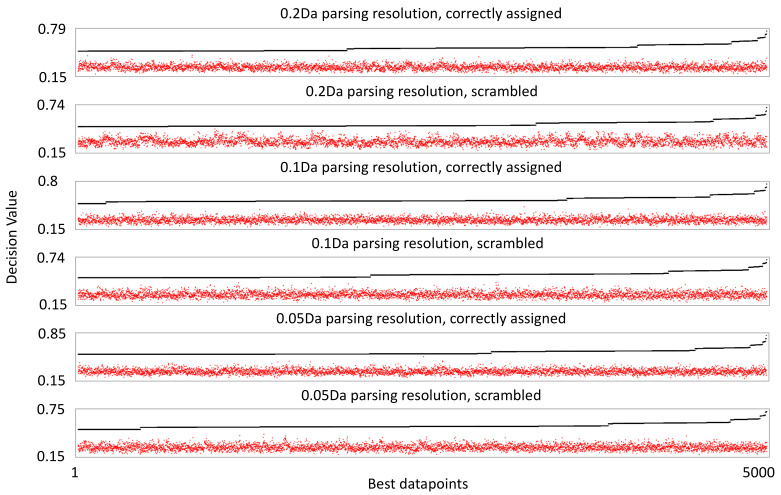
Decision value ranking of mass spectrometry datapoints. The black line shows the scores of the 5000 best scoring datapoints by decision value. The red dots indicate the decision value score when individual values within the corresponding datapoint are randomly linked to a breast cancer stage classification, demonstrating what decision value could be achieved from random sorting of the datapoint values.

We processed the mass spectra from the 39 validation samples using machine learning-based COMPASS software, version 1.1. This allows comparative analysis of the entire MS1 scan data from every subject. We performed comparison based on early and late-stage classification, as for the study performed using a standard workflow. During parsing, in which data are extracted from the mass spec image, we set the COMPASS datapoint size parameters at 40 s on the retention time axis and 0.2 (initial), 0.1 (medium), or 0.05 (High) Da in the *m*/*z* axis. This yielded 684,843, 1,328,207, or 2,540,403 datapoints for analysis at each respective resolution after those containing no mass spec information were discarded. Ranking these datapoints by decision value (DV), an algorithm that measures the separation of values within a datapoint in relation to the biological classification, yields scores with a maximum of 0.79 and 257 datapoints with a score above 0.6 for the initial resolution. Slightly better DV scores are achieved as the resolution is increased, but the number of datapoints with scores above 0.6 increases more dramatically, to 439 at medium resolution and 560 at high resolution. Since no datapoint exhibited DV scores of 1 or greater, it means that there is an overlap of datapoint values when the values are separated by early and late-stage categories. Nonetheless, these DV scores far exceed what can be achieved when the values for the same datapoints are not correctly matched to categories (Figure 2).

We sought to be even more thorough in determining the confidence with which we could assess the dataset. We performed an identical analysis using a mock assembly file, in which the assignment to outcome categories had been randomized. When datapoints were ranked by DV, the highest score was 0.74, and 82 scored above 0.6. Importantly, these datapoints stand out above what can be achieved by randomizing the values within each datapoint with respect to breast cancer stage classification, highlighting potential concerns that information extracted using correctly assigned data is a result of overfitting natural variation in the data. Increasing the resolution had no effect on the best DV scores with the mock assembly file, though more datapoints scored above 0.6—144 for medium resolution and 276 at high resolution.

**Figure 3:**
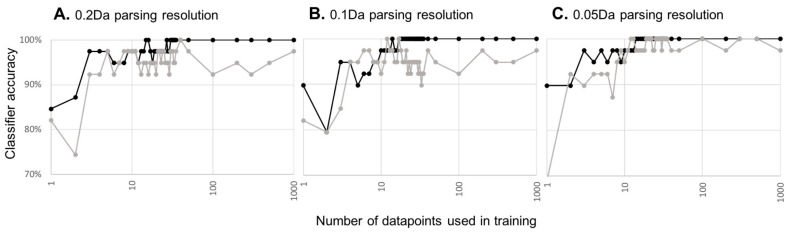
Machine learning accuracy as a function of the amount of training data. Black lines and points indicate machine learning accuracy with correctly assigned data, while grey lines indicate machine learning accuracy derived from the mock dataset. Graphs are for machine learning applied to datapoints generated at 0.2 (**A**), 0.1 (**B**), or 0.05 Da (**C**) parsing resolution along the *m*/*z* axis of the mass spec image.

We then applied the COMPASS’ machine learning analytical module to the correctly assigned and mock datasets using the datapoints generated using initial, medium, and high-resolution parsing described above. COMPASS uses a machine learning algorithm that provides each datapoint a weighted vote. Vote weighting is based on DV, so higher DV datapoint votes count for more than lower DV datapoint votes. This algorithm is less prone to overfitting when applied to small numbers of examples, as is the case for most biological studies. We set COMPASS’ parameters such that 20% of the data was withheld from classifier training and then used to test the classifier’s accuracy. Classifier training and testing were performed in 50 iterations, such that each subject would be withheld from training multiple times and in various combinations. We varied the number of datapoints to be used by the classifier, scanning a range of up to 1000 datapoints (Figure 3). A low number of datapoints yields a high error rate, which is not surprising given the low DV scores for this dataset. As more datapoints are included in machine learning training, the accuracy increases and appears to asymptote to some maximum. Regardless of parse resolution, the correctly assigned dataset reaches 100% accuracy. We do not claim perfect accuracy only that machine learning correctly classifies all of the small number of examples we provide. Importantly, the small number of subjects used in this study limits the precision of the accuracy calculation. With increased parsing resolution and a concomitant increase in the number of datapoints assessed for information content, it requires fewer datapoints to reach maximum machine learning reliability, likely a reflection on small gains in DV achieved with higher parsing resolution.

Can similar classifier accuracy be achieved solely from natural variation in the data? When machine learning is used against the mock assembly file, the same overall accuracy trend is achieved. Few datapoints give a high error rate, which decreases to some minimum as more datapoints are supplied to the classifier. As parsing resolution/total number of datapoints assessed increases, the asymptote occurs earlier and appears to reach a lower minimum. In short, increasing the parsing resolution becomes self-defeating as opportunities increase for detecting false signals from natural variations in the dataset.

Taken together, machine learning can successfully yield a highly accurate classifier with a relatively small study while attempting to detect a subtle biological difference between samples. The caveat is that, depending on the total number of datapoints considered, accuracy and risk of overfitting both emerge with increasing numbers of datapoints. Fortunately, there is a wide range in which the classifier appears to have maximum accuracy with a performance difference that is dramatically different than that generated from natural variation alone.

Cluster analysis, plotting individual datapoint values on a grid with subjects clustered on one axis and datapoints clustered on the other, reveals two general observations (see Data Availability Statement). First, two of the early subject samples behaved markedly differently than all others. They may be outliers representing biologically genuine subsets or may represent quality control issues for these samples. Second, the vast majority of datapoints exhibit upregulation in late-stage samples.

Finally, we selected the top 100 datapoints at each resolution and re-parsed these regions at higher resolution (10 s on the retention time axis and 0.01 Da on the *m*/*z* axis) to ascertain mass spectrometry properties of features within these datapoints more accurately. The resulting lists, one generated for each parsing resolution, contained the mass MS image coordinates for only 3 of the 65 features identified by the standard workflow and reported in Table 1, namely serial numbers 1, 47, and 51, all of which are downregulated in late-stage breast cancer patient samples. Despite a low rate of rediscovery, the lists of high-resolution features generated from workbooks with different initial parsing resolutions contain many of the same features; 48, 35, and 70% of the features generated at initial, medium, and high original parsing resolution, respectively, are unique to their corresponding workbook. Such lists could prove invaluable in a subsequent targeted molecular identification campaign since the list is restricted to regions of the mass spectrum that contain features whose expression correlates highly with important breast cancer stage differences.

## 4. Discussion

The accurate detection of lymph node infiltration in breast cancer patients could inform treatment decisions. Our goal was to evaluate whether a mass spectrometry-based serum biomarker discovery campaign conducted with a small patient cohort could yield models that reliably differentiate between early and late-stage (stage I/II vs. stage III) breast cancer patients. The serum processing and instrumentation approach used here has proven successful in previous studies that sought to predict preterm birth [12] and pre-eclampsia [10] and diagnose Alzheimer’s disease [11].

The approach used here is ‘feature-first’, in that it emphasizes differences detected in MS1 scan spectra and is agnostic to molecular identity of species correlating with disease or physiological states. We took two approaches to analyzing the mass spectra. First, we followed the standard approach conducted in the previously cited studies, which required a large manual component for data alignment, feature validation for low abundance signals, feature measurement, and classical statistical analysis. Second, we employed a computational approach designed to identify differences in mass spectra between groups of samples, assess their relevance to biology by machine learning, and validate the predictive power of the resulting models using algorithmic tools. Both resulted in the successful identification of differentially expressed features within the mass spectra that could be combined to generate reliable predictive models to classify patients by breast cancer stage based on serum analysis.

According to observations based on tumor/stroma interactions, freely circulating factors (tumor-derived serum factors, or TDSFs) are released by the primary tumor and increase with disease progression [6]. While the machine learning-based analysis delivered datapoints that mostly exhibit this expected expression change, the standard approach delivered differentially expressed biomarkers are down-regulated in stage III sera. What might then drive biomarker down-regulation in stage III patients? Perhaps invasive cells cause certain peptides and proteins to become depleted from serum. Invasion is associated with high levels of surface protease expression, whose activity might deplete specific peptides from circulation. Alternately, perhaps anti-invasive species must be depleted for productive colonization of sites distant from the primary tumor. Currently there is scant published evidence for either idea.

We conducted a very small targeted biomarker identification campaign, which focused on biomarkers identified using the standard workflow. The generation of reliable MS2 scans was complicated by peaks near and overlapping that of the targeted precursor ion, as well as by low abundances, leading to incomplete fragment series. However, we did identify a biomarker whose expression is restricted to early-stage breast cancer: a peptide fragment of fibrinogen α chain (FGA).

Fibrinogen is a blood glycoprotein with alpha, beta, and gamma chains. Following vascular injury, thrombin cleaves fibrinogen to release fibrinopeptide A [21], a predominant component of blood clots. In clot degradation, or fibrinolysis, fibrinogen is further cleaved by plasmin into smaller fragments. The fragment identified here, corresponding to amino acids 231–237, is not derived from known thrombin- or plasmin-mediated proteolysis. Thus, the biological context that results in the differential expression of this peptide remains unknown.

Fibrinogen and its fragments play essential roles in regulating cellular processes, such as adhesion, diffusion, vasoconstriction, and chemotaxis, and acting as mitogens for various cell types. Studies have reported connections among cancer progression, prognosis, and survival with fibrin/fibrinogen [22,23,24].

The scale of the data—the number of potential biomarkers evaluated—directly impacts the accuracy limits of multimarker models or algorithms designed to distinguish between sample groups. Since biomarkers are typically both rare and imperfect, larger pools of potential biomarkers are required to build reliable classifiers for the subtle biological differences, such as differences in breast cancer stage. Discovery efforts based on small sample numbers struggle to distinguish real signals from false ones.

Large manual screening of 7500 individual mass spec features for those that correlate with breast cancer stage yields 65 validated candidates, a discovery rate of roughly 1 in 100. Many did not survive confirmation and validation but surviving candidates can be combined to produce 4–6 component multimarker models with moderate accuracy. Meanwhile, a machine-learning-based approach that analyzes the entirety of the MS spectrum, yielding 0.65–2.5 million MS datapoints for analysis depending on resolution settings, produces a machine learning algorithm that approaches 100% classifier accuracy from high information datapoints. The machine learning classifier comprises information from far more independent datapoints than the manual approach, which appears necessary, as classifier training must balance the benefits of increased accuracy with more training data against the emergence of spurious signals from natural variation that also increases with increased training data.

## 5. Conclusions

In this study we characterize the differential expression of low molecular weight, low abundance serum species and develop a multi-marker model that indicates breast cancer stage. Despite the small sample sizes used here, the results are encouraging as proof of principle for the approach, whether using standard or machine learning-based workflows.

## Figures and Tables

**Table 1:** Details of significant biomarkers identified in the discovery study. Markers are defined by their *m*/*z* and charge status. *p*-value is calculated using the marker intensities following normalization, with the overall mean calculated across all the samples. Marker status describes the level of the marker as the disease progresses. Abbreviations: D—downregulated in stage III; U—up-regulated in stage III.

Serial #	Marker *m*/*z*	Charge (z)	*p*-Value	Marker Status
1	403.23	2	0.008	D
2	409.2	2	0.04	D
3	414.23	2	0.003	D
4	415.67	2	0.01	U
5	421.22	2	0.002	D
6	425.25	2	0.002	D
7	430.28	1	0.01	D
8	436.24	2	0.004	D
9	442.23	2	0.001	D
10	443.23	2	0.01	D
11	447.26	2	0.012	D
12	450.3	1	0.007	D
13	451.2	2	0.006	D
14	458.25	2	0.004	D
15	459.24	1	0.03	U
16	464.31	1	0.03	U
17	465.74	2	0.014	D
18	472.3	1	0.007	D
19	473.21	2	0.029	D
20	482.29	1	0.005	D
21	487.25	2	0.003	D
22	488.29	1	0.017	D
23	494.3	1	0.006	D
24	495.23	2	0.014	D
25	497.26	1	0.006	D
26	522.28	1	0.03	D
27	524.26	2	0.01	D
28	537.27	1	0.03	D
29	541.29	1	0.01	D
30	543.31	3	0.019	D
31	546.27	2	0.004	D
32	555.27	1	0.0002	D
33	568.27	2	0.001	D
34	571.25	1	0.0014	D
35	572.63	3	0.008	D
36	575.33	1	0.04	D
37	582.3	2	0.03	U
38	585.3	1	0.006	D
39	590.3	2	0.007	D
40	599.29	1	0.002	D
41	605.28	2	0.03	D
42	629.3	1	0.003	D
43	643.3	1	0.003	D
44	649.32	2	0.01	D
45	656.33	2	0.008	D
46	673.33	1	0.0018	D
47	687.35	1	0.002	D
48	700.36	2	0.003	D
49	709.4	1	0.02	U
50	713.47	1	0.02	U
51	717.35	1	0.002	D
52	721.37	2	0.005	D
53	721.42	1	0.008	U
54	722.37	2	0.002	D
55	731.37	1	0.002	D
56	743.37	2	0.02	D
57	744.3	2	0.04	U
58	747.3	1	0.02	D
59	761.37	1	0.004	D
60	792.58	1	0.019	U
61	819.43	1	0.003	D
62	835.38	1	0.005	D
63	863.43	1	0.002	D
64	879.43	1	0.015	D
65	923.45	1	0.0085	D

**Table 2:** Biomarkers emerging from confirmation study. 425.25a is marked with * to differentiate it from another distinct marker with the same *m*/*z* but exhibiting a different charge state and different elution time.

ID	*m*/*z*	Sensitivity	Specificity	AUC
1	497.26	0.79	0.64	0.79
2	923.45	0.65	0.66	0.72
3	761.37	0.63	0.7	0.7
4	425.25a *	0.66	0.66	0.69
5	722.37	0.58	0.6	0.66
6	585.3	0.59	0.55	0.65
7	458.25	0.55	0.64	0.65
8	747.3	0.67	0.63	0.63
9	555.27	0.63	0.6	0.63
10	442.23	0.59	0.7	0.62
11	546.27	0.56	0.56	0.62
12	879.43	0.5	0.56	0.61

**Table 3:** Multi-marker model performance. Performance and composition of five multi-marker models, including sensitivity, specificity, and area under the curve (AUC) scores in the confirmation (training set, top) and validation studies (test set, bottom). Component numbers refer to the corresponding biomarker ID in Table 2.

Multi-Marker Model	Components	Sensitivity	Specificity	AUC
A	1–2, 5–6	0.875	0.915	0.915
		0.286	0.875	0.84
B	1–2, 4–6	0.858	0.783	0.919
		0.286	0.875	0.76
C	1–2, 5–7	0.858	0.75	0.913
		0.429	0.875	0.804
D	1–3, 5–6	0.875	0.742	0.912
		0.429	0.875	0.77
E	1–6	0.917	0.758	0.915
		0.429	0.875	0.77

**Table 4:** Fragment Ion Calculator results. Sequence DLVPGNF, pI: 3.7998, monoisotopic masses.

Seq	#	B	Y	# (+1)
D	1	116.03481	761.38342	7
L	2	229.11888	646.35648	6
V	3	328.18729	533.27242	5
P	4	425.24005	434.204	4
G	5	482.26152	337.15124	3
N	6	596.30444	280.12978	2
F	7	743.37286	166.08685	1

## Data Availability

The computer scripts, data, and output files are publicly available from https://osf.io/37r5v. Files containing the mass spectrometry data generated for this report are part of a pending submission to PRIDE (www.ebi.ac.uk/pride). Interactive, read-only COMPASS workbooks containing the machine learning analysis reported in this study can be accessed at https://sandbox.mag-bio.com/ with the project title AlZaabi2024.

## Supplementary Materials

The following supporting information can be downloaded at: https://www.mdpi.com/article/10.3390/cancers16132365/s1, Supplementary Table S1: Demographic and clinical characteristics of patients whom serum acquired in the study; Supplementary Table S2. Cross-validation results for classification algorithms. Area under the ROC curve (AUC) using the training data for classification algorithms we compared. We performed 10 iterations of two-fold cross validation and averaged the AUC results across the folds and iterations. Results are shown for up to three markers. When we found that adding a marker did not improve predictive performance, we halted the analysis; therefore, there is no result for Support Vector Machines and three markers.
